# Risk factors associated with pregnancy loss after single euploid blastocysts transfer

**DOI:** 10.3389/fendo.2024.1461088

**Published:** 2025-01-29

**Authors:** Xiaohui Dong, Juanzi Shi, Xiaohua Liu, Danmeng Liu, Wei Li, Xiaoli Zhao, Xia Xue

**Affiliations:** Assisted Reproduction Center, Northwest Women’s and Children’s Hospital, Xi’an, China

**Keywords:** art, frozen embryo transfer (FET), preimplantation genetic testing, risk factor, euploid blastocyst, pregnancy loss

## Abstract

**Objective:**

To detect primary factors influencing pregnancy loss(PL) following the transfer of euploid blastocysts?

**Design:**

Retrospective cohort study.

**Methods:**

To identify potential factors influencing PL, we analyzed multiple variables in both the PL group and the live birth group. In order to minimize the impact of confounding factors, various variables were included in a binary logistic regression analysis.

**Results:**

The PL rate after the transfer of euploid embryos is 13.3% (36 cases of PL out of 270 cases of single embryo transfer). Compared to the live birth group, the PL group had lower E2 levels (2376.03 ± 1553.25 vs 3412.88 ± 2116.47, P=0.007), higher LH levels (2.66 ± 2.47 vs 1.96 ± 1.52, P=0.023) on trigger day of fresh cycle, fewer retrieved oocytes (10.08 ± 5.01 vs 12.77 ± 7.20, P=0.044), higher female BMI (23.26 ± 3.27 vs 22.05 ± 2.69, P=0.016), higher proportion of maternal smokers (50% vs 20.51%, P<0.001), and more day 6 blastocyst transfers ((38.89% vs14.53, P=0.001). Logistic regression analysis revealed that higher LH levels(OR=1.304, 95%CI=1.054-1.613, P=0.015), low E2 levels (OR=0.438, 95%CI=0.242-0.794, P=0.007) on trigger day of fresh cycle, maternal smoking(OR=4.574, 95%CI=1.974-10.601, P<0.001), and day 6 blastocyst transfer(OR=4.610, 95%CI=1.907-11.141, P=0.001) appeared to be associated with increased risk of PL following the transfer of euploid embryos.

**Conclusions:**

Maternal smoking, day of blastocyst transferred, estradiol (E2) and Luteinizing hormone (LH) levels on trigger day of corresponding fresh cycle for transferred blastocysts are all associated with PL following transfer of euploid embryos.

## Introduction

Pregnancy loss (PL) is defined as the spontaneous termination of a pregnancy before fetal viability (1). PL can cause significant harm to women, including adverse psychological and physiological effects. It has been reported that approximately 8% to 20% of recognizable natural pregnancies end in PL (2, 3).

PL has been shown to be associated with an increased risk of various physiological and psychological disorders. Studies suggest that PL is linked to subsequent mental health disorders such as depression, anxiety, and others (4). Furthermore, mothers who experience PL often receive little social attention, further complicating their situation. Additionally, studies suggest a close association between PL and the development of type 2 diabetes (5), cardiovascular diseases (6), and even malignant tumors (7), although the evidence is not yet conclusive. Hence, there is a strong desire among both patients and healthcare providers for a method to predict and prevent PL.

Researches have investigated the factors that impact the rate of PL. Many studies have explored the factors contributing to PL, including maternal age, obesity, embryonic factors, hormone levels during ovarian stimulation, but have not reached consistent conclusions. In recent years, some studies have started to explore the mechanisms underlying PL at deeper levels, such as genes (8, 9), cytokines (10), miRNA (11), and so on. This also provides a broader perspective for more effective research on the mechanisms of PL.

The etiology of early miscarriage is commonly attributed to chromosomal abnormalities, while the reasons for PL following transfer of euploid blastocysts remain unclear. The aim of this study is to investigate the factors influencing the rate of PL after ART treatment in order to better understand its mechanisms. In this study, we aimed to establish an image based on multiple risk factors to effectively predict the probability of patients experiencing PL, thus offering robust evidence for clinical consultation.

## Materials and methods

### Study design

This study was a single-center retrospective cohort study. It was conducted in the Assisted Reproduction Center, Northwest Women’s and Children’s Hospital in Xi ‘an, Shaanxi Province, China and was approved by the ethics committee of the Northwest Women’s and Children’s Hospital (number 2023003). Patients who underwent frozen-thawed embryo transfer (FET) after PGT-A between2017 and 2022 were included. The data were extracted from the electronic medical record system (Wuhan Huchuang, Co., Ltd.; version 9.2.5.8).

### Patients

The study enrolled patients who met the following criteria: (1) The initial cycle of thawing and transferring an euploid embryo following PGT-A; (2) Women under 38 years old. The following cycles were excluded: (1) Patients suffering from uterine infertility caused by conditions such as intrauterine adhesions, endometrial polyps, uterine fibroids, and congenital uterine malformations; (2)Patients with hypertension, diabetes, and systemic autoimmune diseases; (3) Women with ectopic pregnancies, molar pregnancies, continuing pregnancies, and induced abortions.

From 2017 to 2022, according to the inclusion and exclusion criteria, a total of 352 patients were enrolled. Among them, 278 cases resulted in clinical pregnancies. After excluding 8 cases with incomplete clinical data information, a total of 270 patients were included in the study. Ultimately, 234 cases resulted in live births, while 36 cases experienced PL. Patients were divided into the live birth group and the PL group.

### IVF Procedures

For ovarian stimulation, patients receive recombinant and/or urinary gonadotropin therapy in a long protocol with gonadotropin-releasing hormone (GnRH) agonist or GnRH antagonist. In this study, the patients underwent intracytoplasmic sperm injection (ICSI) for fertilization. The embryos were cultured in the 40 μL G1(Vitrolife, Sweden) medium. The embryos were assessed on the third day following the methodology described in our previous study. If the criteria were met, extended culture would be performed to the blastocyst stage. On the 5th and 6th day, according to the Gardner criteria (12), embryos would be graded. Embryos with an expansion grade of 4 or higher, and inner cell mass and trophectoderm grade of C or higher, would undergo biopsy and be cryopreserved by vitrification. The vitrification was performed according to the standard procedure using the Cryotop, an open system (Kitazato BioPharmaCo, Japan).

### Blastocyst morphology

Blastocyst morphological analysis was conducted on the 5th day and 6th day following ICSI fertilization. The content of this article only included the expanded blastocysts, encompassing stages 4, 5, and 6. The evaluation of the inner cell mass (ICM) and trophectoderm (TE) was mainly determined by the cell count and the level of compactness in their organization. The tight packing of numerous cells of ICM/TE was identified as “A”. The clustering of several cells together was identified as” B”. Loose arrangement of a few cells was identified as” C”. In this study, top-quality grade blastocysts referred to those with a grade of BB and above, including AB, BA, BB, and AA. The remaining blastocysts were considered lower quality, including BC, CB, AC, CA, and CC grades.

### FET protocols

The endometrial preparation was performed using the natural cycle or the hormonal treatment cycle. During a natural menstrual cycle, follicles and hormone levels were monitored to identify the timing of spontaneous ovulation or to induce ovulation using a 1000IU hCG injection. The blastocyst transfer procedure was scheduled for day 6 post-ovulation. In the hormonal cycle, oral E2 valerate (Progynova; Bayer Schering Pharma AG) with a dose of 6mg/day was administered from the 5^th^ day of menstrual cycle. Once the endometrial thickness reaches 7-8mm as detected by ultrasound monitoring, the administration of 60mg of progesterone daily for luteal support should commence until a negative hCG levels was obtained or until the 8th week of pregnancy. The blastocyst transfer is typically scheduled on the 7th day of progesterone initiation.

### Outcome measurement

The collected data were analyzed according to the following sections: 1.The population information of two cohorts were analyzed, comprising the age, BMI, and infertility status of the female subjects. 2. Compared the ovarian stimulation variables between two groups of patients during the fresh cycle, such as hormone concentrations on trigger day, initial and total amount of gonadotropin (Gn), etc. 3. Analyzed the impact of various factors on PL.

Clinical pregnancy is defined as the presence of a gestational sac on early ultrasound examination, with or without fetal heart activity. Live birth was characterized by the birth of a viable infant after 28 weeks of gestation post embryo transfer. PL refers to the clinical pregnancy that ultimately did not result in a live birth.

### Statistical analysis

All data analysis was conducted using the Statistical Package for the Social Sciences (SPSS) software, version 26. Continuous variables were expressed as the mean ± standard deviation and a normality test was conducted. Variables conforming to a normal distribution are evaluated using the Student’s t-test, whereas variables deviating from a normal distribution are assessed using the Mann-Whitney U test. Categorical variables were depicted as counts and proportions, and the chi-square test was employed to assess the differences. A multivariate logistic regression was conducted to evaluate the risk factors that affecting PL. The results were presented as odds ratios (ORs) and 95% confidence intervals (CIs). A P-value of less than 0.05 indicated a statistically significant difference.

## Results

We compared of population characteristics between the live birth group and the PL group (**Table 1**). Our analysis revealed no statistically significant differences in maternal age, paternal age, paternal BMI, infertility duration, antral follicle count, dose of gonadotrophins and basic hormone (E2, FSH, LH) levels between the two groups. A statistically significant difference in maternal BMI was observed between the two groups (*P*=0.016). When comparing manipulations in the fresh cycle, differences were found in E2, LH on the HCG trigger day (*P*=0.007, *P*=0.023) and the number of oocytes retrieved (*P*=0.044) between the two groups.

**Table 1:** Population characteristics of the live birth group and the pregnancy loss group.

Characteristics	Live birth group (n=234)	Pregnancy loss group (n=36)	*P*-value
Maternal age (years)	33.04 ± 3.98	33.17 ± 4.05	0.858
Maternal BMI (kg/m^2^)	22.05 ± 2.69	23.26 ± 3.27	**0.016**
Maternal smoking(%)	48(20.51)	18(50)	**<0.001**
Paternal age (years)	34.48 ± 4.92	34.81 ± 4.76	0.713
Paternal BMI (kg/m^2^)	25.19 ± 3.14	25.39 ± 3.36	0.728
Infertility duration (years)	1.63 ± 2.22	1.75 ± 2.18	0.762
Type of infertility			O.591
primary	48 (20.51)	6 (16.67)	
secondary	186 (79.49)	30 (83.33)	
Antral follicle count	13.31 ± 5.87	12.64 ± 5.86	0.522
Basal LH, IU/L	4.95 ± 3.88	4.69 ± 2.44	0.693
Basal FSH, IU/L	6.06 ± 2.46	6.51 ± 1.99	0.293
Basal E2, pg/mL	28.45 ± 15.10	31.31 ± 19.27	0.311
Dose of gonadotrophins, IU	2357.08 ± 711.02	2558.82 ± 890.66	0.138
Manipulations in the fresh cycle			
Ovarian stimulation regimen			0.725
Agonist (%)	60 (25.64)	7 (19.44)	
Antagonist (%)	162 (69.23)	27 (75.00)	
Other (%)	12 (5.13)	2 (5.56)	
LH on the day of HCG trigger, IU/L	1.96 ± 1.52	2.66 ± 2.47	**0.023**
P on the day of HCG trigger, ng/mL	1.35 ± 0.66	1.23 ± 0.61	0.383
E2 on the day of HCG trigger, pg/mL	3412.88 ± 2116.47	2376.03 ± 155 3.25	**0.007**
No. of oocytes retrieved	12.77 ± 7.20	10.08 ± 5.01	**0.044**
Variables in the FBT			
Endometrial preparation			0.520
Natural cycle	55 (23.50)	6 (16.67)	
Artificial cycle	179 (76.50)	30 (83.33)	
endometrium thickness, mms	10.15 ± 1.66	9.84 ± 1.61	0.296
Day of the blastocyst transferred			**0.001**
Day 5 (%)	200 (85.47)	22 (61.11)	
Day 6 (%)	34 (14.53)	14 (38.89)	
Quality of the blastocyst transferred			0.082
Top quality	164 (89.13)	20 (10.87)	
Lower quality	70 (29.91)	16 (18.60)	

Statistical significant values are highlighted in bold.

As for the variables in the FBT, there existed a statistically significant disparity in the day of blastocyst transfer between the two cohorts, with a notably reduced incidence of PL observed on day5 blastocyst transfer (9.91% vs 29.17, *P*=0.001). No significant differences were observed in the endometrial preparation protocols, endometrium thickness and quality of the blastocyst transferred examined in our study.

**Figure 1:**
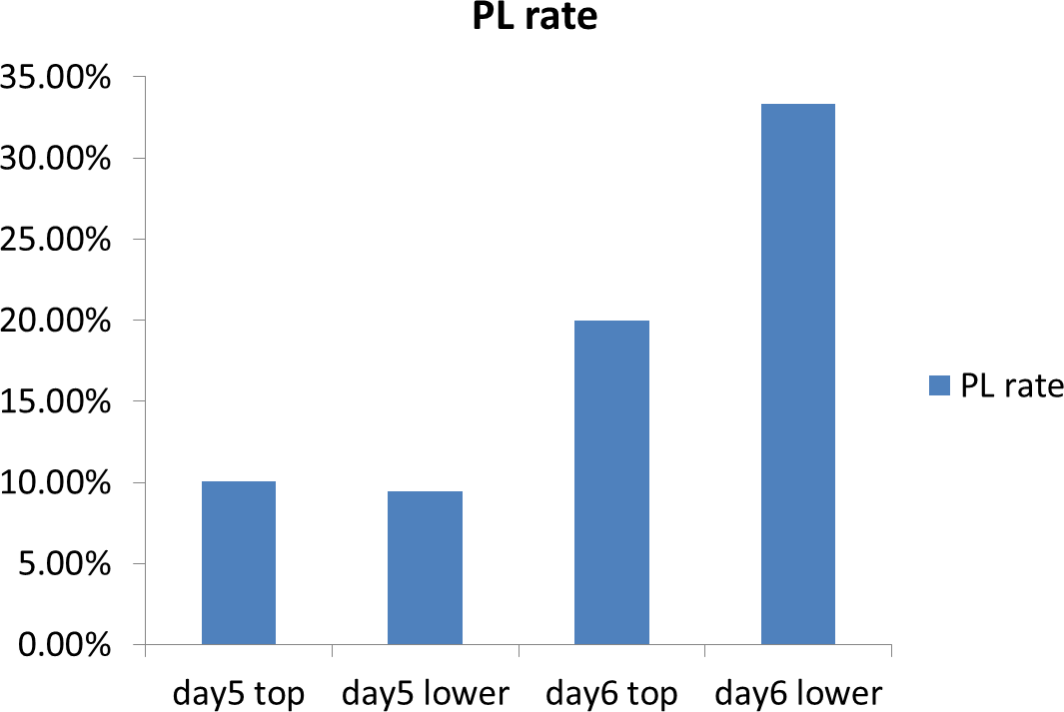
Pregnancy loss (PL) rate stratified by blastocyst day and quality.

In response to practical requirements in real-world applications, we conducted an analysis on the combined impact of blastocyst quality and day of transfer on PL. As shown in **Table 2**, we observed differences in the probability of PL among the four groups (day 5top, day 5lower, day 6top, day 6lower) when we divided the enrolled patients into these groups (*P*=0.003). The corresponding PL rates for the four groups were 10.06%, 9.43%, 20.00%, and 33.33%, as shown in **Figure 1**. The results of logistic regression indicate that compared to day 6lower embryos, the day 5top and day 5lower groups may potentially reduce the occurrence rate of PL. The results of logistic regression indicate that compared to day 6lower embryos, the day 5top and day 5lower groups may potentially reduce the occurrence rate of PL (day 5top: OR=0.224, 95%CI=0.093-0.539, *P*=0.001; day 5lower: OR=0.208, 95%CI=0.065-0.672, *P*=0.009) (**Table 3**).

**Table 2:** Comparison of the day and score amongst the live birth group and the pregnancy loss group.

	N	Live birth	Pregnancy loss	*P*-value
				0.003
day 5 top	169	152 (89.94)	17 (10.06)	
day 5 lower	53	48 (90.57)	5 (9.43)	
day 6 top	15	12 (80.00)	3 (20.00)	
day 6 lower	33	22 (66.67)	11 (33.33)	

Statistical significant values are highlighted in bold.

**Table 3:** Logistic regression model of blastocyst day and score with regard to risk of pregnancy loss.

	OR(95%CI)	*P*-value
day 6 lower	Reference	
day 5 top	0.224(0.093-0.539)	**0.001**
day 5 lower	0.208(0.065-0.672)	**0.009**
day 6 top	0.500(0.116-2.148)	0.351

In odds ratios, bold indicates significant difference.

A logistic regression analysis was conducted based on **Table 1** to explore various factors associated with PL. The results were presented in **Table 4**. We could see that a lower E2 and higher LH levels on the trigger day of the fresh cycle corresponding to the blastocysts transferred appeared to be associated with an increased risk of PL (LH: OR=1.304, 95%CI=1.054-1.613, *P*=0.015; E2: OR=0.438, 95%CI=0.242-0.794, *P*=0.007). Simultaneously, transferring a day 6 blastocyst (OR=4.610, 95%CI=1.907-11.141, *P*=0.001) and maternal smoking (OR=4.574, 95%CI=1.974-10.601, *P*<0.001) also had been shown to increase the risk of PL.

**Table 4:** Logistic regression model of PL after transfer of euploid blastocyst.

	OR(95%CI)	*P*-value
Maternal age (year)	0.987 (0.885-1.100)	0.815
Maternal BMI (kg/m^2^)	1.108 (0.966-1.272)	0.144
Paternal age (years)	1.038 (0.921-1.170)	0.541
Paternal BMI (kg/m^2^)	0.981 (0.866-1.111)	0.758
LH on the day of HCG trigger, IU/L	**1.304 (1.054-1.613)**	**0.015**
E2 on the day of HCG trigger, pg/mL	**0.438 (0.242-0.794)**	**0.007**
endometrium thickness, mms	0.854 (0.672-1.087)	0.201
Day6 blastocyst transferred	**4.610 (1.907-11.141)**	**0.001**
Maternal smoking	**4.574 (1.974-10.601)**	**<0.001**

In odds ratios, bold indicates significant difference.

## Discussion

Based on the findings of this study, higher levels of LH and lower levels of E2 on the trigger day of the fresh cycle, day 6 blastocyst transfer and maternal smoking appeared to elevate the risk of PL in cycles involving the transfer of euploid blastocysts.

The documented incidence of PL in natural conceptions varies from 8% to 20% (2, 3, 13). It is estimated that approximately 30% of pregnancies result in miscarriage, with some cases potentially going undetected (13, 14). Because of the unique characteristics of the ART population, this proportion is expected to be higher. Cai et al (15) showed that among the population undergoing IVF, the PL rate for patients with PCOS was 23%, compared to 21.9% for non-PCOS patients. Therefore, we speculate that there may be certain factors during the IVF process that could increase the risk of PL. Chromosome imbalance, also known as aneuploidy, is the primary factor leading to PL in humans (16). In this study, patients underwent transferring with euploid embryos, allowing for a more precise examination of the impact of factors other than the embryos on PL.

Numerous studies have been conducted to investigate potential factors contributing to PL. Reports indicated a strong correlation between maternal age and the incidence of PL, including both early and late miscarriages (17, 18). Our study did not observe a significant alteration in the rate of PL as maternal age increased. This was easy to understand, which means that age alone may not be the determining factor for PL when all transferred embryos were euploid. A study conducted by Spandorfer et al. (19) revealed that among PLes in women over 40 years old, 82% were attributed to chromosomal abnormalities in the fetus, compared to 65% in younger women. This also aligns with the perspective that aneuploidy serves as the predominant factor leading to PL in women of advanced age (20).

With regard to gamete or embryo manipulation, prior investigation indicated that the type of ovarian stimulation protocols used and human chorionic gonadotropin (HCG) levels on the 14th day are associated with PL (21). Our research results suggest that there is no correlation between ovarian stimulation protocols and HCG levels on the 14th day post-transfer with PL. Nevertheless, lower levels of E2 and higher levels of LH on the trigger day could potentially heighten the likelihood of PL.

The impact of LH levels post controlled ovarian stimulation (COS) on assisted reproductive technology (ART) outcomes remains a topic of debate. Benmachiche et al. (22) discovered that higher levels of LH were associated with increased live-birth rate and reduced PL rate. Similarly, according to Westergaard et al. (23), a LH concentration <0.5 on the 8th day of COS significantly reduces the success LBR and increases the risk of early miscarriage. However, some study (24) have indicated that the levels of LH on trigger day do not have an impact on live birth rates (LBR) and miscarriage rates. LH plays a crucial role in the development of ovarian follicles. Could elevated levels of LH potentially have a detrimental effect on oocyte quality, consequently raising the likelihood of PL? This warrants further investigation.

E2, as the most potent estrogen, is synthesized by ovarian granulosa cells using androstenedione and testosterone as precursors (25). In COS, the level of E2 on the trigger day can reflect the quantity of retrieved oocytes. In our study, we found a significant correlation between lower levels of E2 and PL. The dose of Gn in the PL (PL) group was comparable to that in the live birth group, and in some cases even higher in the PL group (although not statistically different). However, the PL group had a lower number of retrieved oocytes and lower E2 levels, reflecting a poorer response to ovarian stimulation in patients in the PL group. This may indicate the responsiveness of the patient’s ovaries to stimulant drugs, which may predict the probability of subsequent PL.

The incidence of aneuploidy increases with prolonged blastulation duration [16], leading to an elevated rate of miscarriages (26). It has been shown that the miscarriage rate of day 6 blastocysts is higher than that of day 5 blastocysts transferred (27). When the transferred blastocysts were all euploid, the results were also inconsistent. In our study, the miscarriage rate of blastocysts on day 6 was significantly higher (29.17% vs 9.91%). After including various confounding factors such as material/paternal age, BMI, pre-transfer endometrial thickness, maternal smoking, and number of miscarriages in a binary logistic regression analysis, this difference still exists. This is consistent with the majority of previous research findings (28). nevertheless, a study Abdala et al. (29) demonstrated comparing clinical outcomes of euploid blastocysts of day5 and day6 transferred found no difference in miscarriage rates. The discrepancy may stem from variations in the criteria employed by different centers for the inclusion of biopsiable blastocysts.

We also found that maternal smoking can significantly increase the incidence of PL. A study based on genetic data conducted by Yuan et al. (30) indicated a potential causal relationship between maternal smoking and miscarriage. In our study, after controlling for embryo-specific factors, maternal smoking as an independent factor significantly increased the rate of PL. Consistent with our research, a well-established negative correlation between maternal smoking and miscarriage has been identified (31–33). Therefore, it is essential to implement preconception counseling and public health initiatives targeting women of reproductive age to discourage smoking, particularly during pregnancy.

The main limitation of this study is attributed to the small sample size of euploidy blastocyst transfers, leading to inadequate statistical power to discern any disparities in the live birth and the PL group. Due to the limitation of sample size, we did not stratify the analysis based on the quality of the blastocysts for further investigation. This limitation should be addressed in future studies.

In conclusion, after the transfer of euploid blastocysts, there are still factors such as higher levels of LH and lower levels of E2 on the trigger day, day6 blastocyst transfer and smoke independently contribute to PL.

## Data Availability

The raw data supporting the conclusions of this article will be made available by the authors, without undue reservation.

## References

[1] Bender Atik R Christiansen OB Elson J Kolte AM Lewis S Middeldorp S. Eshre guideline: recurrent pregnancy loss. Hum Reprod Open. (2018) 2018:hoy004. doi: 10.1093/hropen/hoy004 31486805 PMC6276652

[2] Rossen LM Ahrens KA Branum AM. Trends in risk of pregnancy loss among us women, 1990-2011. Pediatr perinatal Epidemiol. (2018) 32:19–29. doi: 10.1111/ppe.12417 PMC5771868 29053188

[3] Linnakaari R Helle N Mentula M Bloigu A Gissler M Heikinheimo O. Trends in the incidence, rate and treatment of miscarriage-nationwide register-study in Finland, 1998-2016. Hum Reprod. (2019) 34:2120–8. doi: 10.1093/humrep/dez211 31747000

[4] Delgado L Cobo J Giménez C Fucho-Rius GF Sammut S Martí L. Initial impact of perinatal loss on mothers and their partners. Int J Environ Res Public Health. (2023) 20:1304. doi: 10.3390/ijerph20021304 PMC9858910 36674059

[5] Egerup P Mikkelsen AP Kolte AM Westergaard D Rasmussen S Knop FK. Pregnancy loss is associated with type 2 diabetes: A nationwide case-control study. Diabetologia. (2020) 63:1521–9. doi: 10.1007/s00125-020-05154-z 32424542

[6] Peters SAE Yang L Guo Y Chen Y Bian Z Tian X. Pregnancy, pregnancy loss, and the risk of cardiovascular disease in chinese women: findings from the China kadoorie biobank. BMC Med. (2017) 15:148. doi: 10.1186/s12916-017-0912-7 28784170 PMC5547470

[7] Mikkelsen AP Egerup P Ebert JFM Kolte AM Nielsen HS Lidegaard Ø. Pregnancy loss and cancer risk: A nationwide observational study. EClinicalMedicine. (2019) 15:80–8. doi: 10.1016/j.eclinm.2019.08.017 PMC6833468 31709417

[8] Hromadnikova I Kotlabova K Krofta L. First-trimester screening for miscarriage or stillbirth-prediction model based on microrna biomarkers. Int J Mol Sci. (2023) 24:10137. doi: 10.3390/ijms241210137 PMC10299132 37373283

[9] Maksiutenko EM Barbitoff YA Nasykhova YA Pachuliia OV Lazareva TE Bespalova ON. The landscape of point mutations in human protein coding genes leading to pregnancy loss. Int J Mol Sci. (2023) 24:17572. doi: 10.3390/ijms242417572 PMC10743817 38139401

[10] Spathakis M Filidou E Pappa C Arzou BC Georgiadis A Kontomanolis EN. Spontaneous abortion is associated with differentially expressed angiogenic chemokines in placenta and decidua. Arch Gynecol Obstet. (2023) 308:821–30. doi: 10.1007/s00404-022-06725-8 35997970

[11] Galaziou A Filidou E Spathakis M Arvanitidis K Arzou BC Galazios G. Imbalance of growth factors mrna expression associated with oxidative stress in the early pregnancy loss. J maternal-fetal neonatal Med. (2022) 35:6150–6. doi: 10.1080/14767058.2021.1907337 33820497

[12] Gardner DK Lane M Stevens J Schlenker T Schoolcraft WB. Blastocyst score affects implantation and pregnancy outcome: towards a single blastocyst transfer. Fertil Steril. (2000) 73:1155–8. doi: 10.1016/s0015-0282(00)00518-5 10856474

[13] Wang X Chen C Wang L Chen D Guang W French J. Conception, early pregnancy loss, and time to clinical pregnancy: A population-based prospective study. Fertil Steril. (2003) 79:577–84. doi: 10.1016/s0015-0282(02)04694-0 12620443

[14] Griebel CP Halvorsen J Golemon TB Day AA. Management of spontaneous abortion. Am Family physician. (2005) 72:1243–50. 16225027

[15] Cai H Mol BW Gordts S Wang H Wang T Li N. Early and late pregnancy loss in women with polycystic ovary syndrome undergoing ivf/icsi treatment: A retrospective cohort analysis of 21 820 pregnancies. BJOG: an Int J obstetrics gynecology. (2021) 128:1160–9. doi: 10.1111/1471-0528.16590 33142019

[16] Gruhn JR Zielinska AP Shukla V Blanshard R Capalbo A Cimadomo D. Chromosome errors in human eggs shape natural fertility over reproductive life span. Sci (New York NY). (2019) 365:1466–9. doi: 10.1126/science.aav7321 PMC7212007 31604276

[17] Lidegaard Ø Mikkelsen AP Egerup P Kolte AM Rasmussen SC Nielsen HS. Pregnancy loss: A 40-year nationwide assessment. Acta Obstet Gynecol Scand. (2020) 99:1492–6. doi: 10.1111/aogs.13860 32255196

[18] Bu Z Hu L Su Y Guo Y Zhai J Sun YP. Factors related to early spontaneous miscarriage during ivf/icsi treatment: an analysis of 21,485 clinical pregnancies. Reprod BioMed Online. (2020) 40:201–6. doi: 10.1016/j.rbmo.2019.11.001 31883882

[19] Spandorfer SD Davis OK Barmat LI Chung PH Rosenwaks Z. Relationship between maternal age and aneuploidy in *in vitro* fertilization pregnancy loss. Fertil Steril. (2004) 81:1265–9. doi: 10.1016/j.fertnstert.2003.09.057 15136087

[20] Verdyck P Altarescu G Santos-Ribeiro S Vrettou C Koehler U Griesinger G. Aneuploidy in oocytes from women of advanced maternal age: analysis of the causal meiotic errors and impact on embryo development. Hum Reprod. (2023) 38:2526–35. doi: 10.1093/humrep/dead201 37814912

[21] Hu L Du J Lv H Zhao J Chen M Wang Y. Influencing factors of pregnancy loss and survival probability of clinical pregnancies conceived through assisted reproductive technology. Reprod Biol Endocrinol. (2018) 16:74. doi: 10.1186/s12958-018-0390-6 30086781 PMC6081896

[22] Benmachiche A Benbouhedja S Zoghmar A Humaidan P. Low lh level on the day of gnrh agonist trigger is associated with reduced ongoing pregnancy and live birth rates and increased early miscarriage rates following ivf/icsi treatment and fresh embryo transfer. Front Endocrinol (Lausanne). (2019) 10:639. doi: 10.3389/fendo.2019.00639 31620091 PMC6759793

[23] Westergaard LG Laursen SB Andersen CY. Increased risk of early pregnancy loss by profound suppression of luteinizing hormone during ovarian stimulation in normogonadotrophic women undergoing assisted reproduction. Hum Reprod. (2000) 15:1003–8. doi: 10.1093/humrep/15.5.1003 10783342

[24] Luo X Li L Lin N Ma R Li Y Wu Z. Low endogenous lh on the cos initiation day of a gnrh-agonist regimen increases the risk of early pregnancy loss and adverse art outcomes. Front Endocrinol (Lausanne). (2022) 13:830567. doi: 10.3389/fendo.2022.830567 35265040 PMC8898906

[25] Wu CH Kuo TC Wu HH Yeh GP Tsai HD. High serum estradiol levels are not detrimental to *in vitro* fertilization outcome. Taiwan J Obstet Gynecol. (2007) 46:54–9. doi: 10.1016/s1028-4559(08)60108-4 17389191

[26] Tiegs AW Sun L Patounakis G Scott RT. Worth the wait? Day 7 blastocysts have lower euploidy rates but similar sustained implantation rates as day 5 and day 6 blastocysts. Hum Reprod. (2019) 34:1632–9. doi: 10.1093/humrep/dez138 31402381

[27] Park DS Kim JW Chang EM Lee WS Yoon TK Lyu SW. Obstetric, neonatal, and clinical outcomes of day 6 vs. Day 5 vitrified-warmed blastocyst transfers: retrospective cohort study with propensity score matching. Front Endocrinol (Lausanne). (2020) 11:499. doi: 10.3389/fendo.2020.00499 32849288 PMC7418454

[28] Boynukalin FK Gultomruk M Cavkaytar S Turgut E Findikli N Serdarogullari M. Parameters impacting the live birth rate per transfer after frozen single euploid blastocyst transfer. PloS One. (2020) 15:e0227619. doi: 10.1371/journal.pone.0227619 31929583 PMC6957140

[29] Abdala A Elkhatib I Bayram A Arnanz A El-Damen A Melado L. Day 5 vs day 6 single euploid blastocyst frozen embryo transfers: which variables do have an impact on the clinical pregnancy rates? J Assist Reprod Genet. (2022) 39:379–88. doi: 10.1007/s10815-021-02380-1 PMC8956773 35064434

[30] Yuan S Liu J Larsson SC. Smoking, alcohol and coffee consumption and pregnancy loss: A mendelian randomization investigation. Fertil Steril. (2021) 116:1061–7. doi: 10.1016/j.fertnstert.2021.05.103 34187701

[31] Pineles BL Park E Samet JM. Systematic review and meta-analysis of miscarriage and maternal exposure to tobacco smoke during pregnancy. Am J Epidemiol. (2014) 179:807–23. doi: 10.1093/aje/kwt334 PMC3969532 24518810

[32] Farioli A Curti S Violante FS Mattioli S. Smoking and miscarriage risk. Epidemiol (Cambridge Mass). (2010) 21:918. doi: 10.1097/EDE.0b013e3181e57008 20924241

[33] Ghimire PR Akombi-Inyang BJ Tannous C Agho KE. Association between obesity and miscarriage among women of reproductive age in Nepal. PloS One. (2020) 15:e0236435. doi: 10.1371/journal.pone.0236435 32760090 PMC7410243

